# Report of the Committee on the Case of Montrose A. Pallen

**Published:** 1865-07

**Authors:** 


The committee appointed to prepare an answer to the protest in the case of Dr. Pallen, reported as follows, through its chairman, Dr. Hunt:—

We, the undersigned, beg leave to recommend the following reply to the protest of certain members of the American Medical Association against the action of the said Association in the case of one Montrose A. Pallen.

We remark, first, as to the right of protest in an association of this kind, that it is one which needs scarcely ever to be exercised, and the use of which is in general a bad precedent. When a motion is fairly before a body, is carefully considered, and then deliberately acted upon, that submission to law and to a majority which is important to be cultivated as a principle, and which is particularly needed to be exemplified by prominent men as the grand lesson taught by the times, should lead the minority to be content to express their dissent in the usual method. With due deference to those who signed the protest, we cannot but express our regret that, while in the course of years many points have been discussed before this body, involving direct medical interests, and yet have been left to the decisions of the majority, so far as we know, the first case calling forth a protest is in the behalf of a man, in reference to whom we had the distinct statement of Dr. C. C. Cox, of Maryland, that he, early in the war, left St. Louis, in the interests of the rebellion, and that the testimony in the case referred to was explicit in reference to his complicity with the attempted crime. We at least exercise no right not already granted to these protestants, in expressing our dissent from the propriety of their action in not submitting to the decision of this body after the question was once discussed, and then twice after reconsidered and the former action reaffirmed, and that too in the absence of the gentlemen who were the movers of the resolution. Secondly, we beg leave to notice the fact that the protestants have erred as to matters of fact. The Association has in no way formally declared that no sufficient proof had been adduced of the guilt of Montrose A. Pallen. The fact that the word proved was amended by the insertion of the words "has been declared under oath," is merely the difference between the legal issuing of a case and that kind of evidence which is entirely satisfactory and conclusive to even a guarded public sentiment. It was on this evidence and on the statements and opinions of members, that the Association saw fit to base their action. They felt that the course of Pallen, previous to this averred act, and the testimony in the case, were sufficient grounds for their action. There are times when a man may be known by the company he keeps, and in such a barbarous wickedness as the one involved, the whole testimony and evil association justify us in this action.

If the case was one that could be tried by us, so much the better; but the grounds of suspicion are so enormous, as in our view to justify our course of action. We are not injuring him, for the cloud already upon him is one to which ours will not add a feather. We are but protecting ourselves. When the evidence of mistake is shown, we will gladly acknowledge our error and retrace our course. But is it not best to separate from our Association one who has been in intimate association with the vilest enemies of humanity? We have high ideas of personal rights, but one who has been in leagued friendship with those making the boldest attack on the dearest personal rights of universal humanity cannot complain, until his character is vindicated, that he is cut off from association with those who by profession, as well as by their manhood, are philanthropists and patriots. While we agree to the general idea that no man shall be condemned and punished until his guilt is established, we look upon guilt as having degrees, and as sometimes being established, independent of the decision of the courts; and in cutting him off from the Association, we do not recognize ourselves as "condemning and punishing him" in the legal and opprobrious sense in which those terms are used when one is consigned to the prison or the gallows; but as protecting ourselves from the odium of association with one who, we have evidence enough to satisfy us, has chosen as associates not only rebels, but assassins, and who, while he is unable to clear his skirts, is not particularly jeopardized in life, liberty, or the pursuit of happiness, by our act.

The President then announced that he had received the following telegraphic despatch from Montrose A. Pallen:—

MONTREAL, CANADA EAST, JUNE 9.

*To the President of the National Medical Association:*—I have been expelled from the association upon unrebutted testimony for participating in a crime which I execrate as much as any gentleman in the convention. I will produce affidavits to prove Sandford Conover's evidence to be infamous perjury. I beg a reconsideration of the vote, and that it be referred to a committee before whom I pledge myself to still preserve the fair fame belonging to every member of the association. MONTROSE A. PALLEN.

A motion was made to accept and adopt the report of the committee as the sense of the association. The motion was discussed at some length, and finally laid upon the table.

A special committee on insanity was appointed, consisting of Drs. Alfred Hitchcock, of Massachusetts; Isaac Ray, of Rhode Island; S. H. Tewksbury, of Maine; B. F. Barker, of New York; and J. S. Butler, of Connecticut.

The Secretary read the following resolution offered by Dr. Garrish, of New York:—

*Resolved*, That the American Medical Association, with heartfelt and national pride, extend to the Army and Navy Surgeons their thanks for the prompt manner in which they have met the calls and perils of the battle field, demanded by our country.

*Resolved*, That we tender to the families and friends of those who have sacrificed their lives in the cause of humanity, our earnest sympathies for the bereavement the have sustained. They have restored to us and the whole nation a peaceful and happy home for all future time.

The resolutions were adopted.

On motion, the duty of revising the list of permanent members for republication was referred to the committee on medical necrology.

Dr. Burns, of Pennsylvania, presented the following resolutions, which were unanimously adopted:—

*Resolved*, That the American Medical Association, in closing their annual session in the city of Boston, June 9th, 1865, do most sincerely tender a vote of thanks to their medical brethern, the Governor of Massachusetts, city authorities and inhabitants of Boston, for their very kind and generous hospitalities to the assembled delegates from all parts of our country; and in biding them farewell we shall ever cherish the remembrance of their kindness, and express the hope that prosperity, with every domestic, social, intellectual, and religious blessing may be their abiding portion.

By Dr. Kennedy of New York, resolutions were offered as follows:—

*Resolved*, That the thanks of the American Medical Association be and are hereby tendered to His Honor Mayor Lincoln and the authorities of the city of Boston, for the kind reception and splendid entertainment given to the association during their stay in the city.

*Resolved*, That the thanks of the association are due, and are hereby presented, to the committee of arrangements of the association for the faithful and satisfactory manner in which they have discharged the arduous duties imposed upon them.

*Resolved*, That the thanks of the association be presented to our president, Dr. N. S. Davis, for the singularly able and impartial manner in which he has discharged the duties of his office.

The following was offered by Dr. Van Kleek, of New York:—

*Resolved*, That this is a National Association, and as, in its earlier days, we enjoyed with pleasure and profit the intercourse with our professional brothers of all parts of our country, and as the unhappy feud which for four years divided the nation, has now ceased and peace has again come, we trust forever, —so we hope soon again to meet our members and delegates from the South on this platform of fraternalization, and to this end we extend to them an earnest invitation and promise them a cordial welcome.

The following by Dr. Bowditch was offered by Dr. Hibberd:

*Resolved*, That this association has learned with deep regret that there are fears of famine and consequent disease and pestilence occurring in some parts of Georgia and South Carolina, passed over by Gen. Sherman's army in its victorious course.

*Resolved*, That this association would urge upon the Sanitary and Christian Commissions to send agents to learn the exact facts, and, if need be, to take prompt and efficient measures to prevent or mitigate such a distressing result.

The following was offered by Dr. Bronson:—

*Resolved*, That the thanks of this association be and they are hereby tendered to Major John Morrisey, the Sergeant-at-Arms of the Massachusetts Legislature, and his assistants, for the very able and efficient manner in which they have performed the functions of their offices during the sessions of the association.

The resolutions were all unanimously adopted.

The venerable Dr. Child made a short address, speaking as follows:—

I feel extremely gratified that I am permitted on behalf of the State Medical Society to be present on this occasion, and I have also been extremely gratified in the proceedings of the association; and, permit me to say, very much so as respects the management of the presiding officer [cheers]; and I would unite in expressing the thanks of the association for the worthy manner in which he has performed his services, and I hope a reply will be heard from him on this parting occasion. [Applause.]

